# Case Report: A Possible Case of Congenital Erythropoietic Porphyria in a Gir Calf: A Clinical, Pathological, and Molecular Approach

**DOI:** 10.3389/fvets.2021.632762

**Published:** 2021-03-12

**Authors:** Cintia Regina Rêgo Queiroz, Mizael Machado, Cristiana Raach Bromberger, Jose Paes Oliveira-Filho, Alexandre Secorun Borges, Benito Soto-Blanco, José Renato Junqueira Borges, Antônio Carlos Lopes Câmara, Márcio Botelho de Castro

**Affiliations:** ^1^Veterinary Pathology Laboratory, University of Brasília, Campus Darcy Ribeiro, Brasília, Brazil; ^2^Instituto Nacional de Investigación Agropecuaria, Tacuarembó, Uruguay; ^3^Department of Veterinary Clinical Science, School of Veterinary Medicine and Animal Science, São Paulo State University (UNESP), Botucatu, Brazil; ^4^Department of Veterinary Clinics and Surgery, Veterinary College, Universidade Federal de Minas Gerais, Belo Horizonte, Brazil; ^5^College of Agronomy and Veterinary Medicine, Large Animal Veterinary Teaching Hospital, University of Brasília, Brasília, Brazil

**Keywords:** Zebu, cattle, porphyrin, pink teeth, uroporphyrin

## Abstract

Congenital erythropoietic porphyria (CEP) is a rare hereditary autosomal recessive disease which has never been reported in Zebu cattle. A 3-day-old Gir calf showed teeth discoloration, fever, dehydration, and dyspnea. The main gross findings were pink-colored teeth, red-brown periosteum and bone marrow, and a fluorescent bright pink coloration of the bone marrow and articular surfaces under ultraviolet light. Aggregates of periodic acid-Schiff (PAS)-stained porphyrin pigments were evident in the lungs, kidneys, and the liver. An intron 8 single-nucleotide polymorphism (SNP) in both the Gir calf and control animals, along with the absence of the uroporphyrin III synthetase (*UROS*) gene mutation, was observed. Most SNPs were located in the intron regions of the *UROS* gene without relevance for CEP. A continuous loss of genetic variability and an increase in inbreeding in some herds may be related to CEP in Gir cattle, one of the most prominent Zebu breeds worldwide. In summary, this study describes a presumptive case of CEP in a Gir calf based on clinical and pathological findings. A definitive diagnosis would require the measurement of porphyrin levels in blood, urine, or tissues or the identification of *UROS* gene defects.

## Background

Porphyrias result from the deficiency of an acquired, or a hereditary, enzyme, which is involved in the synthesis of the heme group (1). Congenital erythropoietic porphyria (CEP), known as pink tooth or Gunther's disease, is a rare hereditary autosomal recessive disease which has been reported in pygmy hedgehog, cattle, pigs, cats, foxes, rats, and human (2–4). CEP is related to a genetic defect in porphyrin metabolism in association with a deficiency of the uroporphyrin III synthetase (*UROS*) enzyme, which is required for the biosynthesis of the heme group (1). *UROS* deficiency leads to the accumulation of uroporphyrin I and coproporphyrin I (pathogenic porphyrins) (3, 5), which are released into the bloodstream, subsequently followed by their deposition in bones, teeth, and skin and the excretion through urine and feces (2, 5). The accumulation of porphyrin results in pink or red-brown coloration of the bones and teeth and a bright pink fluorescence under ultraviolet (UV) light (1, 2, 6) and the possibility of the urine turning red when exposed to sunlight (2), which may also lead to hemolytic anemia, photosensitization, and a low growth rate (2, 7–9).

Congenital bovine erythropoietic porphyria is a rare global disease in high-inbred herds (4) and has been reported in Shorthorn (10), Longhorn, Holstein-Friesian, Jersey, Hereford, Angus, Ayrshire, and Jamaican cattle breeds, with a higher incidence in females (4, 7). Here, we report the clinical, pathological, and molecular findings of the first likely case of CEP in a Gir calf (*Bos taurus indicus*).

## Case Description

A 3-day-old female Gir dairy calf presented with apathy and a reduced sucking reflex. Physical examination revealed fever, dehydration, dyspnea, recumbence, gastrointestinal hypomotility, and discoloration of the pink-marked teeth. There was no information regarding genetic diversity and inbreeding rates in the herd. The evaluation of thoracic radiographs revealed alveolar lung patterns suggestive of aspiration pneumonia.

Hematologic analysis showed leukocytosis [34,900 cells/μl; reference interval (RI): 7,600–11,800 cells/μl] with neutrophilia (23,034 cells/μl; RI: 2,200–9,800 cells/μl) and left shift (5,584 cells/μl; RI: 0–100 cells/μl) (11, 12), indicating inflammation attributable to the clinical diagnosis of bronchopneumonia. No significant changes were detected in red blood cell data or morphology. Blood plasma was clear without signs of hemolysis, and urine had a normal pale-yellow color. In serum biochemical analysis, the only significant abnormalities were low serum gamma-glutamyl transferase activity (76 UI/L: 288–3,170 UI/L), hypoproteinemia (43 g/L; RI: 44–74 g/L), hypoalbuminemia (24 g/L; RI: 26–31 g/L), and hypoglobulinemia (19 g/L; RI: 30–52 g/L) (13, 14). These findings suggest a passive immune transfer failure related to low colostrum ingestion (15) due to a reduced sucking reflex.

During hospitalization, the calf developed cyanotic mucous membranes and was treated with fluid therapy (normal saline solution, IV), broad-spectrum antibiotics (ceftiofur: 5 mg/kg, IV, 7 days), and non-steroidal anti-inflammatory drugs (flunixin meglumine: 2.2 mg/kg, IV, 3 days) for pneumonia. The general condition of the calf remained unstable in the next 4 days, and euthanasia was elected based on ethical considerations. The discoloration of the pink-marked teeth raised the presumptive diagnosis of CEP.

**Figure 1:**
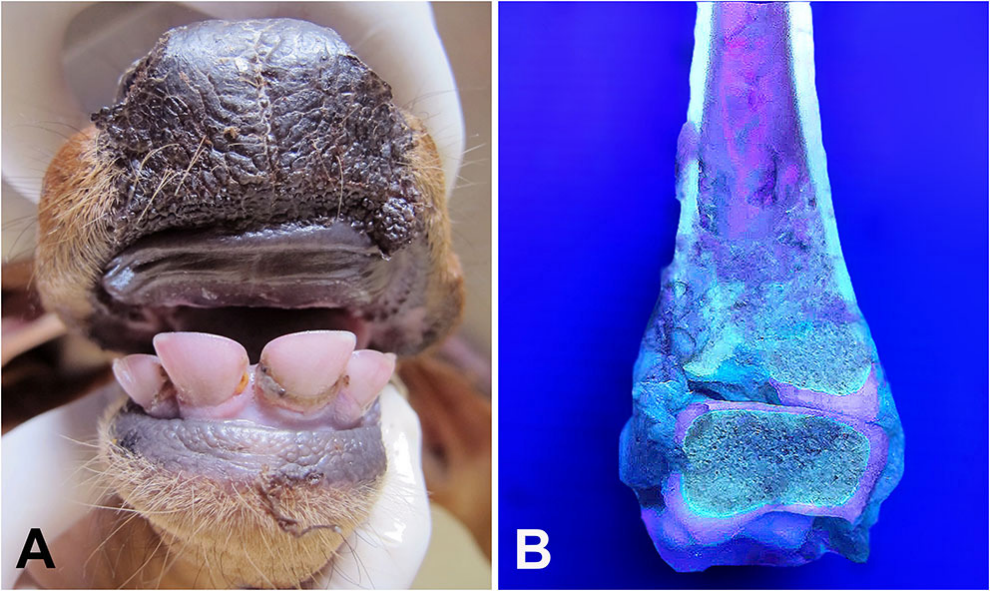
Gir calf. **(A)** Marked pink discoloration of the teeth. **(B)** Bight pink-red fluorescent color of bone marrow and cartilages viewed under ultraviolet (UV) light.

**Figure 2:**
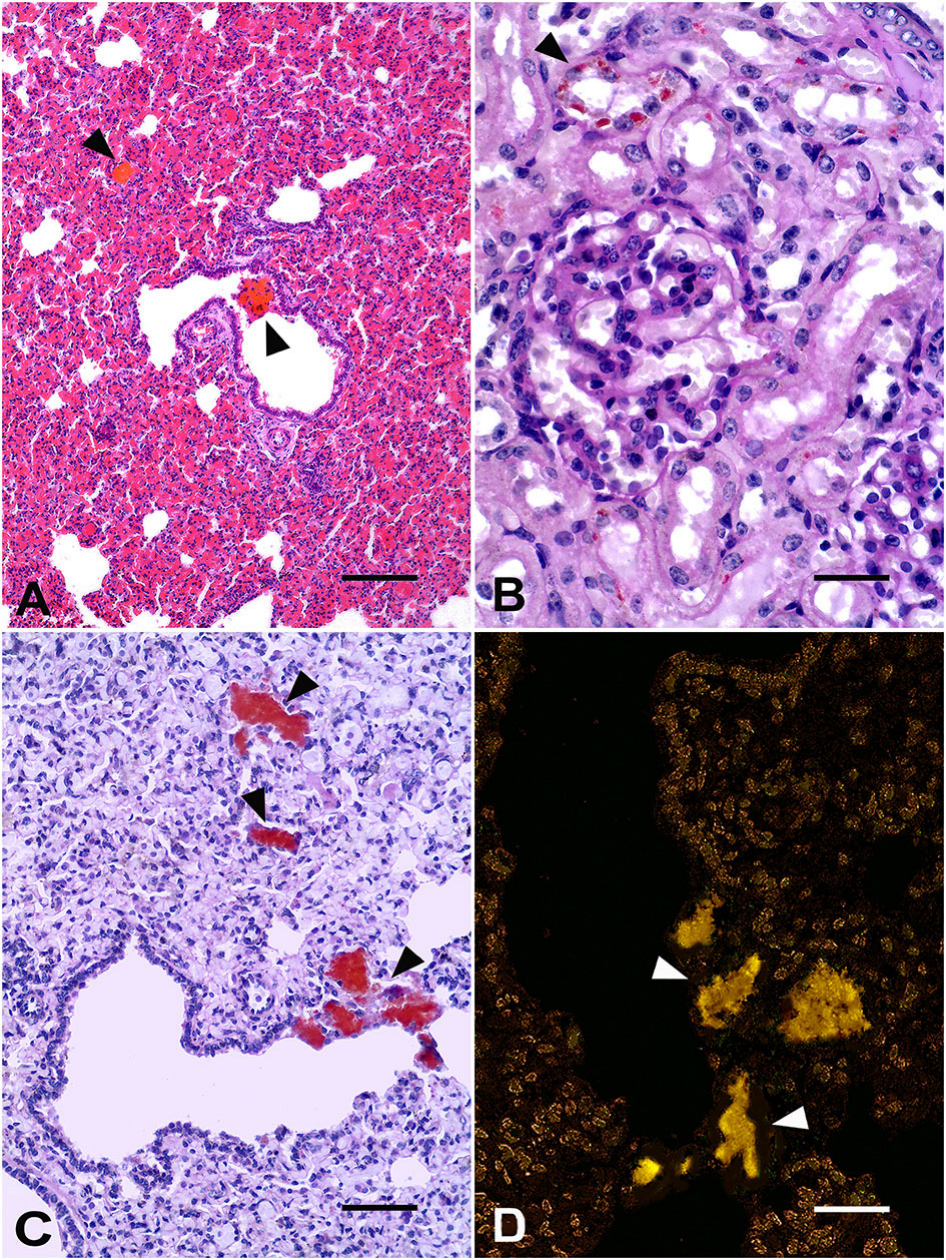
Gir calf. **(A)** Lung: deposits of brown-orange porphyrin pigments (arrowheads) in the bronchiolar lumen. H&E, Bar = 50 μm. **(B)** Kidney: orange-brown granules of porphyrin within the cytoplasm of renal tubule epithelial cells (arrowhead). Periodic acid-Schiff (PAS) stain, Bar = 25 μm. **(C)** Lung: deposits of porphyrin in the alveoli and pulmonary septa and interstitium (arrowheads). PAS stain, Bar = 25 μm. **(D)** Lung: orange-yellow spontaneous fluorescence of porphyrin aggregates (arrowheads). Unstained lung, fluorescence microscopy, Bar = 25μm.

Marked pink discoloration of the teeth (Figure 1A) and red-brown periosteum and bone marrow of long bones were the most significant gross findings on postmortem examination. After exposure to UV light, the bone marrow and articular surfaces demonstrated a fluorescent bright pink-red color (Figure 1B). The lungs had cranioventral consolidation, and the kidneys were slightly enlarged and demonstrated a diffused red-brown color. No skin lesions were observed. At the microscopic level, the presence of mild multifocal deposits of brown-orange porphyrin pigments in the pulmonary septa and interstitium, bronchiolar epithelium, and lumen (Figure 2A), as well as in hepatocytes and the proximal renal tubule epithelium, was observed. No other gross or microscopic findings were detected in other organs or tissues.

The staining of periodic acid-Schiff (PAS) leads to strong staining of porphyrin deposits in the epithelial cells of renal tubules (Figure 2B) and in the pulmonary alveoli, epithelium, and bronchiolar lumen (Figure 2C). Porphyrin deposits showed negative Perls Prussian blue staining. Additionally, porphyrin deposits showed an orange-yellow spontaneous fluorescence in the unstained lung (Figure 2D) and kidney sections under a fluorescence microscope (spectral transmission: 550–610 nm) (2, 16). Aspiration bronchopneumonia with moderate, multifocal neutrophilic infiltrates associated with an amphophilic fluid material inside the alveoli was also detected. Thus, a neonate calf with pink teeth, a fluorescent bright pink-red color of bone marrow and articular surfaces under the exposure of UV light, and the microscopic evidence of porphyrin deposits in different tissues enabled the diagnosis of a probable case of CEP (2, 4, 17, 18).

As a previous study has suggested that a mutation in the bovine *UROS* gene may cause CEP (6), 9 coding *UROS* exons were sequenced using DNA from the liver of the CEP calf and the blood of 4 control animals. Animals used as controls (three Gir and one Brown Swiss) were healthy and unrelated to the CEP calf. PCR was performed using primers (Table 1) as previously described Agerholm et al. (6), and specific primers were designed to amplify the 9 coding *UROS* exons and the 5′ and 3′ intron–exon junctions. PCR products were used in a sequencing reaction, and sequences and electropherograms of the affected calf and control animals were compared with the *Bos taurus* uroporphyrinogen III synthase sequence deposited in GenBank™ (Gene ID: 613425).

**Table 1:** PCR and sequencing primer sets used in this study.

**Primer sets**	**Primer sequences**	**Product (bp^a^)**	**Melting**
Agerholm^b^_UROS_Exon 1	5′- ACTGCCAGGCCATAATGAAG−3′	200	60°C
	5′- GGGGCCTGTTTCACAATTTA−3′		
Agerholm_UROS_Exon 2	5′- GCCAGCTCAAAGCTGTATCC−3′	174	60°C
	5′- GGGAAACCAAAGGTGTCTCA−3′		
Agerholm_UROS_Exon 3	5′- GAAGGATGGATGGATGGATG−3′	333	60°C
	5′- AAGTCACTGCGCTGTTTCAA−3′		
Agerholm_UROS_Exon 4	5′- GCAGCTCACATCGAATTTCA−3′	203	60°C
	5′- GGGCCAGGAATAGGGATAAA−3′		
Agerholm_UROS_Exon 5	5′- TAAAGCACCTGCCTTCGTCT−3′	194	60°C
	5′- CCGGGGCCCTACTCTACTTA−3′		
Agerholm_UROS_Exon 6	5′- CCACACCCCTGACACGTTAC−3′	235	60°C
	5′- GCTCTGGGGTGACACTTTGT−3′		
Agerholm_UROS_Exon 7	5′- CCCTGGGGGACCTCTACTAA−3′	282	60°C
	5′- GCGAGTCACAATCTGCAATG−3′		
JPOF^c^_UROS_Exon 1	5′- CAAGCCCATAAATGCCAAGTC−3′	355	62°C
	5′- CCCACTCCTCAACTGTTAGC−3′		
JPOF_UROS_Exon 3	5′- GTGACCTTAAAGACCTGGAGTAG−3′	347	62°C
	5′- GCATCACCGACGGGATAAA−3′		
JPOF_UROS_Exon 8	5′- GAGCCTCTTTCTCCTTGGTC−3′	253	54°C
	5′- TGGCTTCGCACGGAAAT−3′		
JPOF_UROS_Exon 9.1	5′- CCCTTTCCAGGCTTTGTTCT−3′	294	62°C
	5′- TGGAGCGTCGTCCTGAT−3′		
JPOF_UROS_Exon 9.2	5′- CTGTCTTCTCCCTGCAGTTC−3′	213	62°C
	5′- CAATGCCTGGCTCCACA−3′		

a *Base pairs*,

b *Primers previously described by Agerholm et al. (6)*,

c *Primers designed in the present study*.

The *Bos taurus UROS* gene has 9 introns and 10 exons. One of these exons (non-coding exon) is located in the 5′ untranslated region, whereas the others are coding exons (1 to 9). Alignments of the affected and non-affected CEP cattle-obtained sequences revealed 20 variants (Table 2).

**Table 2:** Variants in the uroporphyrin III synthetase (*UROS*) sequences of those obtained from the affected and nonaffected congenital erythropoietic porphyria (CEP) cattle.

**Variant**		**Location**	**Ref. allele^a^**	**Genotyped alleles**				
				**CEP Gir**	**Gir 1**	**Gir 2**	**Gir 3**	**Brown Swiss**
1	NC_037353.1:c.45.316.150	Intron 1	C	C	C	T	C/T	C
2	NC_037353.1:c.45.316.097	Intron 1	A	A/G	A	G	A/G	A
3	NC_037353.1:c.45.316.064	5′UTR, coding exon 1	A	A/C	A	C	A/C	A
4	NC_037353.1:c.45.315.963	Intron 2	A	A/G	A	G	A/G	A
5	NC_037353.1:c.45.315.775	Coding exon 2	C	C/T	C/T	C/T	C/T	C/T
6	NC_037353.1:c.45.314.234_3	Intron 3	-	- /insT	-	- /insT	- /insT	-
7	NC_037353.1:c.45.314.224	Intron 3	G	G/A	G	G	G	G
8	NC_037353.1:c.45.314.148	Coding exon 3	G	G/A	G	G	G	G
9	NC_037353.1:c.45.314.148	Intron 4	T	T	T/A	T	T	T
10	NC_037353.1:c.45.307.078	Intron 6	G	G/A	G/A	G	G	G
11	NC_037353.1:c.45.307.066	Intron 6	G	G/A	G	G	G	G
12	NC_037353.1:c.45.307.004	Coding exon 6	G	G/A	G/A	G	G	G
13	NC_037353.1:c.45.306.933	Intron 7	G	G/A	G/A	G/A	G/A	G
14	NC_037353.1:c.45.304.870	Coding exon 8	C	C/T	C/T	C/T	C/T	C
15	NC_037353.1:c.45.304.803	Intron 9	G	G/T	G/T	G	G	G
16	NC_037353.1:c.45.304.791	Intron 9	C	C/T	C/T	C/T	C/T	C
17	NC_037353.1:c.45.301.137^b^	Intron 9	G	G	G	G	G	G
18	NC_037353.1:c.45.301.131	Intron 9	A	A/G	A	A/G	A/G	A
19	NC_037353.1:c.45.301.117	Intron 9	G	G	G	G/A	G/A	G
20	NC_037353.1:c.45.301.004	Coding exon 9	T	C	C	C	C	C

a *According to Bos taurus UROS Gene ID: 613425*.

b *Previously described by Agerholm et al. (6)*.

## Discussion

Pink coloration of teeth and bones is a unique and characteristic finding of CEP, distinguishing it from other diseases (19). CEP has been reported mainly in Taurine cattle breeds (4, 6, 7, 10) and has never been reported previously in a Zebu cattle breed. Genealogical studies of some affected familial clusters have demonstrated that CEP is an autosomal disease in the Holstein breed (20). CEP has also been associated with pedigree cattle in which inbreeding or close line-breeding is practised (4, 7, 9, 19), as well as with a higher frequency of consanguineous marriages in human (3). A molecular study also provided evidence that a mutation affecting the bovine ortholog of *UROS* was associated with the disease in a family of Holstein cattle (6). However, sequences of both the CEP calf and control animals (unrelated to the CEP calf) showed the wild-type allele G in homozygosis in the single-nucleotide polymorphism (SNP) located within the spliceosome attachment region in intron 8 of the *UROS* gene (Table 2 Variant 17 NC_037353.1:c.45.301.137), which was previously detected in a lineage of Holstein cattle with CEP (6). In the current study, sequencing of the *UROS* gene showed that most of the SNPs were located in the intron regions and were apparently non-specific to CEP, as they were also present in at least one of the control animals. In addition, 5 synonymous SNPs were found in the coding exons and were identified in both the affected and non-affected CEP cattle.

Gene defects in CEP carriers have been reported in taurine breeds (21), and thus far, genealogical assays of these genes have never been conducted in Zebu breeds. Although the genetic structure of the herd of the Gir calf is unknown, the population structure and inbreeding rates of Zebu cattle in Brazil have shown an increase in total inbreeding in all Zebu breeds, a decrease in ancestors, and the loss of genetic variability resulting in adverse effects of breeding (22). There is an apparent trend for increased rates of inbreeding and decreased effective population sizes in Gir cattle (22). In Gir dairy cattle herds, inbred animals accounted for over 60% of all animals and may have evolved from a narrow genetic base with little genetic variability in Brazil (23). These traits are associated with the adverse effects of inbreeding, such as an increase in autosomal congenital diseases, including CEP.

The CEP calf in the current study was female. Although the inheritance is not sex-linked (21), the incidence of CEP is higher in females (4, 7). The affinity of porphyrins for mineral components usually results in more significant deposition and pigmentation in active mineralization sites of the teeth and bones of young animals (2). Clinical findings can vary between animals with porphyria, depending mainly on the residual levels of the *UROS* activity and the amount of porphyrin deposited in the tissues (3, 5).

Skin injury associated with exposure to the sun was not detected in the Gir calf. The calf was housed in a shelter since birth, which possibly prevented photosensitization. Photosensitization is a non-specific clinical sign commonly observed in CEP; however, its occurrence and intensity are determined by porphyrin deposition levels in the skin and by the level of exposure to the sun. High sun exposure in calves with CEP may not result in photodermatitis if the plasma levels of the porphyrins are low (6, 7). Fever, leukocytosis with neutrophilia, and the left shift due to aspiration bronchopneumonia observed in the calf were not primarily related to the porphyria and were probably caused by suckling difficulty. CEP can compromise the development of an animal, resulting in a lower growth rate than that of other animals of the same age kept under similar management conditions, which in turn can impact the welfare of the animal (1, 24).

Histological findings detected in the Gir calf are not usually described or assessed in the affected cattle and can be typically unremarkable or unrelated to porphyrin deposition (7, 24). Porphyrin aggregates within renal tubular epithelial cells and lungs have previously been reported in other cases in cattle (4) and were similar to those detected in humans and cats with CEP, including the orange-yellow spontaneous autofluorescence under UV light microscopy (16, 25). Porphyrin pigment deposition may be frequent in many human tissues, such as the lungs, heart, adrenal glands, kidneys, liver, spleen, bone marrow, and choroid plexus (25). Mild porphyrin deposition in renal tubular epithelial cells did not cause renal function impairment or damage in the calf, in contrast to the severe porphyrin-associated renal failure reported in cats (16). Serum, urine, and fecal porphyrin levels were not evaluated in the Gir calf; however, they were believed to be low because anemia, hemolysis, and urine color changes were not detected. Hemolytic anemias are usually associated with high serum levels of porphyrin (25). Considering that the staining of PAS porphyrin pigment is not specific, the absence of staining of the iron pigment in the suspected porphyrin aggregates in the tissues (negative Perls Prussian blue staining) strengthens the diagnosis of porphyrin pigment deposition (16, 25) in the calf. Despite the laboratory test limitations of our study, discoloration of the teeth, periosteum, and bone marrow of long bones and bright pink-red fluorescence of bone marrow and articular surfaces under UV light exposure, in addition to microscopic findings, characterized the first likely case of CEP in a Zebu calf.

Although they do not cause the characteristic pink staining of teeth and bones, primary differential diagnoses for CEP include erythropoietic protoporphyria and congenital porphyria in Limousin cattle that have undergone photosensitization (teeth are normochromic in both conditions) (1, 2). Babesiosis and hepatogenous photosensitization should also be considered in the differential diagnoses of CEP (1) and were ruled out in this calf after the laboratory and pathological evaluation. *Dentinogenesis imperfecta* (dentinal dysplasia) is characterized by small and translucent pink-gray teeth (2) and was differentiated grossly and histologically from the pink teeth of the Gir calf with CEP.

Porphyrias are rare and complex diseases with a considerable variation in clinical presentation and lead to a significant impact on animal welfare and life expectancy. The continuous loss of genetic variability and increase in inbreeding in Zebu breeds may be related to CEP. Genetic assays, breeding programs, and mating strategies to avoid inbreeding in the Zebu herd may prevent CEP and other inherited diseases.

## Data Availability Statement

The original contributions presented in the study are included in the article/supplementary material, further inquiries can be directed to the corresponding author/s.

## Ethics Statement

Ethical review and approval was not required for the animal study because the manuscript is a case report of spontaneous disease. Written informed consent was obtained from the owners for the participation of their animals in this study.

## Author Contributions

CQ, MM, BS-B, JB, and AC performed clinical and laboratory evaluations. CQ, MM, CB, JO-F, AB, and MC performed pathological and molecular examinations. MC drafted the manuscript. All authors read and approved the final manuscript.

## Conflict of Interest

The authors declare that the research was conducted in the absence of any commercial or financial relationships that could be construed as a potential conflict of interest.
